# Water-Soluble Molecular Cages for Biological Applications

**DOI:** 10.3390/molecules29071621

**Published:** 2024-04-04

**Authors:** Giovanni Montà-González, Eduardo Ortiz-Gómez, Rocío López-Lima, Guillermo Fiorini, Ramón Martínez-Máñez, Vicente Martí-Centelles

**Affiliations:** 1 Instituto Interuniversitario de Investigación de Reconocimiento Molecular y Desarrollo Tecnológico (IDM), Universitat Politècnica de València, Universitat de València, Camino de Vera s/n, 46022 Valencia, Spain; gmongon@upv.es (G.M.-G.); eortgom@doctor.upv.es (E.O.-G.); glfiorin@doctor.upv.es (G.F.); 2 Departamento de Química, Universitat Politècnica de València, Camí de Vera s/n, 46022 Valencia, Spain; 3 CIBER de Bioingeniería Biomateriales y Nanomedicina, Instituto de Salud Carlos III, 46022 Valencia, Spain; 4 Unidad Mixta de Investigación en Nanomedicina y Sensores, Instituto de Investigación Sanitaria La Fe (IISLAFE), Universitat Politècnica de València, Avenida Fernando Abril Martorell, 106, 46026 Valencia, Spain; 5 Unidad Mixta UPV-CIPF de Investigación en Mecanismos de Enfermedades y Nanomedicina, Centro de Investigación Príncipe Felipe, Universitat Politècnica de València, Avenida Eduardo Primo Yúfera, 3, 46012 Valencia, Spain

**Keywords:** molecular cages, host-guest chemistry, anticancer applications, supramolecular chemistry, metal-organic cages, organic cages

## Abstract

The field of molecular cages has attracted increasing interest in relation to the development of biological applications, as evidenced by the remarkable examples published in recent years. Two key factors have contributed to this achievement: First, the remarkable and adjustable host–guest chemical properties of molecular cages make them highly suitable for biological applications. This allows encapsulating therapeutic molecules to improve their properties. Second, significant advances have been made in synthetic methods to create water-soluble molecular cages. Achieving the necessary water solubility is a significant challenge, which in most cases requires specific chemical groups to overcome the inherent hydrophobic nature of the molecular cages which feature the organic components of the cage. This can be achieved by either incorporating water-solubilizing groups with negative/positive charges, polyethylene glycol chains, etc.; or by introducing charges directly into the cage structure itself. These synthetic strategies allow preparing water-soluble molecular cages for diverse biological applications, including cages’ anticancer activity, anticancer drug delivery, photodynamic therapy, and molecular recognition of biological molecules. In the review we describe selected examples that show the main concepts to achieve water solubility in molecular cages and some selected recent biological applications.

## 1. Introduction

Molecular cages are promising synthetic hosts that mimic encapsulation processes found in nature [1]. One of the key aspects of molecular cages is the preorganization that provides the cage framework, providing enhanced host–guest properties compared to analog macrocyclic structures [2]. Molecular cages encompass two primary categories: metal-organic cages and purely organic cages [3,4]. When guest substrates are confined in the cavity of the cage, they are isolated from the bulk solution [5]. The encapsulation process is highly size- and shape-dependent, providing high specificity and selectivity towards specific guest molecules. In addition, encapsulation produces different effects on the guest molecule depending on the cage properties ranging from protection from the surrounding media to activation of catalytic reactions. These effects result in many different applications of molecular cages, such as catalysis [6,7,8,9], stabilization of chemical species [10,11], and the separation process [12,13,14], among many others [3,4,15,16]. In contrast, biological applications are emerging, and are still in their infancy. This review will show recent examples of cages for biological applications, and an analysis of structural requirements to achieve the solubility of cages in water. We have focused on relevant selected examples that show the high potential of this growing field. The wide range of properties exhibited by molecular cages positions them as promising candidates for sophisticated biological and biomedical applications in the near future.

## 2. Scope of the Review

This review focuses on selected representative examples of water-soluble molecular cages for biological applications, including both metal–organic cages and purely organic cages. We have also included selected examples of non-water-soluble cages to show concepts such as synthetic efficiency or cage formation pathways. Other related relevant molecules to cages are macrocycles such as cyclodextrins and cucurbiturils with the ability to form inclusion complexes with guest molecules, enhancing their solubility for drug delivery applications [17,18,19]. Due to the narrow focus of the review on cages, we have not included cyclodextrins and cucurbiturils in this review. The review focuses on cages with large cavities of sufficient size to encapsulate organic guest molecules. The review focuses on selected examples mainly from the past decade, with a large number of examples from the last five years. The literature search has been included up to the end of 2023.

## 3. Main Concepts

Water-soluble molecular cages are promising host molecules for biological applications. They can selectively encapsulate guest molecules in water including therapeutic drugs, biological molecules, etc. The main challenge to using molecular cages in bio-applications is achieving water solubility and stability. It must be noted that biological media contain numerous components that make it even more challenging to achieve these requirements in comparison to pure water; e.g., inorganic salts present in biological media reduce solubility and also compete for metal coordination in metal–organic cages that may result in cage disassembly. 

### Solubility and Stability in Water

Water solubility depends on the nature of the molecular cage, requiring, in some instances, the modification of its chemical structure with water solubilizing groups. The charged nature of some cages, in both metal–organic cages and pure organic cages, makes it possible to achieve water solubility. In these cases, the solubility can be improved by changing counteranions; for example, using anions such as NO_3_^–^ improves water solubility, whereas hydrophobic PF_6_^–^ anions facilitate solubility in organic solvent. Adding charges can increase the water solubility of molecular cages, but such modifications may also influence the biological effects (e.g., compounds containing negative charges have poor cell membrane penetrability). Therefore, the overall cage charge must be considered as compatible with the target biological application. In contrast, it is in general difficult to achieve water solubility in neutral, purely organic cages that are in general hydrophobic. In these cases, water solubility can be achieved by cage functionalization with water-solubilizing groups such as sulfonates, trimethyl ammonium, carboxylates, long polyethylene glycol chains, etc. 

Moreover, the stability of cages is influenced by the nature of the chemical bonds. The best stability is associated with cages that have inert covalent bonds. This can be achieved using irreversible bond formation at the cage formation step, or by post-synthetic modification of the reversible bonds used in the cage self-assembly (see below). This last type of reaction is widely used, as reversible bond formation enables cage formation with quantitative yields in most cases. Generally, purely organic cages have better stability relative to metal–organic cages because of the higher stability of covalent bonds versus metal coordination bonds. In this general statement, it should be considered that covalent organic cages based on reversible bonds have limited stability. For instance, imine bonds and bororante ester bonds are in general hydrolyzed in water.

## 4. Synthesis

The synthesis of molecular cages requires the assembly of building blocks that must have an appropriate preorganization, i.e., specific shape and geometry, in a similar fashion to macrocycles [20]. The assembly of the building blocks into the cage structure involves both thermodynamic and kinetic aspects that must be considered in the synthetic conditions employed for the cage construction [21]. In this regard, whereas reactions involving irreversible and reversible bond formation can be employed to prepare cage molecules, reversible bonds are widely employed due to their high efficiency [3,4].

**Figure 1:**
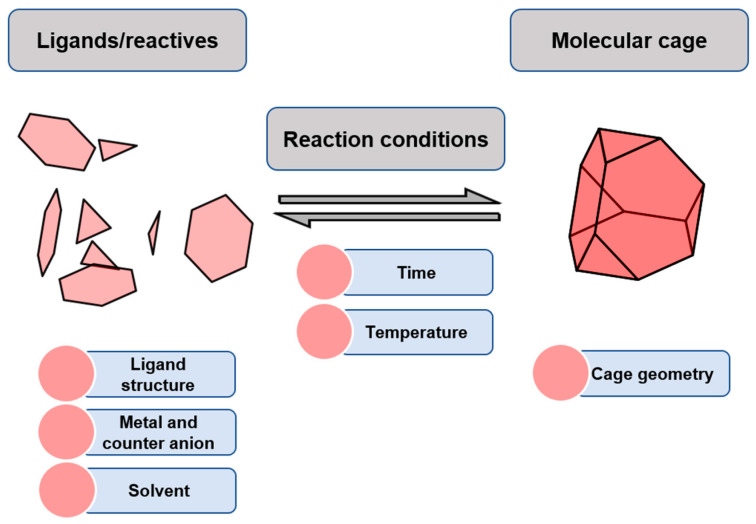
Synthesis of molecular cages through reversible bonds.

The synthesis of metal–organic cages takes place through the self-assembly of ligands and metal centers to form the cage structure under thermodynamic control. The simplicity of this high-yielding method allows the generation of numerous polyhedral architectures from a minimal number of building block components [22]. Furukawa and his team analyzed the synthetic conditions and structural features of metal–organic cages for a very large set of cages reported in the literature (Figure 1). They found that the feasibility of cage formation is determined by the rigidity of the ligands. Most of the analyzed ligands that form cages are rigid. A minor set of flexible ligands also form cages, but they require additional interactions such as template encapsulation or intra-/inter-ligand interactions to favor cage formation. Another key aspect of cage formation is the lability of metal ions, which determines the reaction temperature. For example, cages formed with Pt(II) require more temperature than cages obtained from Pd(II), or cages formed with Rh(II) require more temperature than cages obtained from Cu(II). Coordinative solvents are often used to favor the reversibility of the formed coordination bonds. This facilitates the equilibrium reaction steps that yield cage formation, avoiding kinetically trapped intermediates that hinder cage formation [23].

Similarly, the synthesis of fully organic molecular cages takes place by the self-assembly of ligands to form the cage structure. However, their synthesis can take place through two different bond-forming routes: irreversible bond formation and reversible bond formation (usually known as dynamic covalent chemistry, abbreviated as DCC) [24]. Well-established reversible reactions used to prepare cages include imine/hydrazone, boronic ester, and disulfide bonds [3]. The success of synthesizing molecular cages depends on the specific dynamic covalent bond-forming reaction conditions, as well as the choice of building blocks, which must have a suitable geometry and a favorable preorganization [25]. Here we examine representative examples to show the pros and cons of both irreversible and reversible synthetic methods.

**Figure 2:**
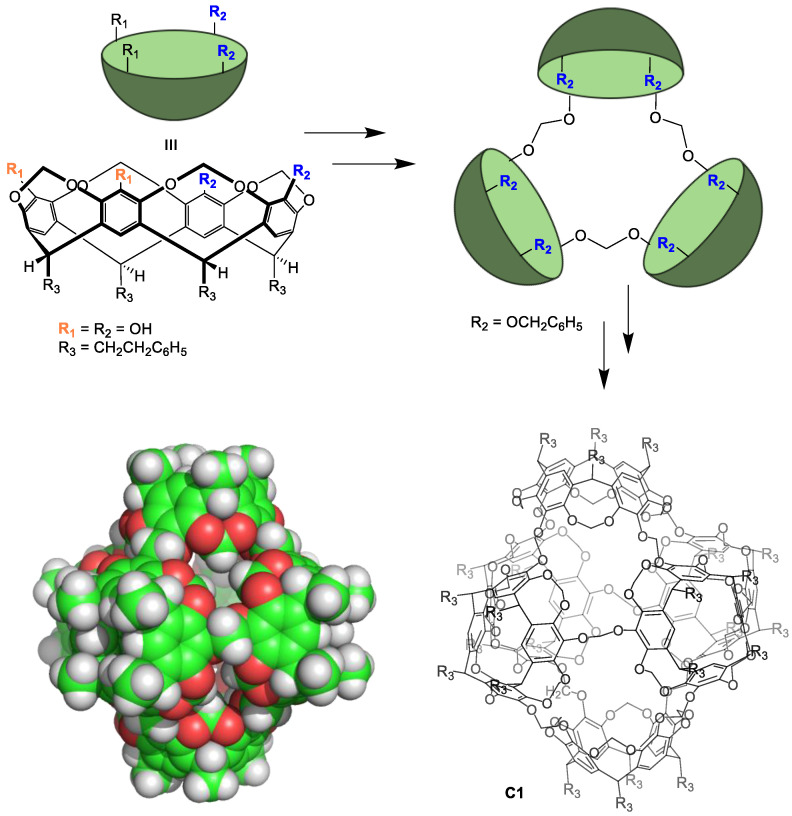
Synthesis of a molecular cage **C1** through irreversible bonds [26].

Preparing non-equilibrium cages using irreversible chemical reactions typically results in low cage formation reaction yields and requires high dilution techniques to obtain the desired cage. In contrast to the lability of metal–organic cages, the resulting cages have excellent chemical and thermal stabilities. The main drawback of irreversible bond formation is that it does not allow for the self-correction of errors occurring during cage formation. This results in obtaining oligomeric byproducts that reduce the cage formation yield. A representative example by Sherman and coworkers shows the synthesis of a cage compound with six cavitand building blocks. These components are held together by irreversible bonds in four lineal reaction steps from the starting materials. The yield of each step was 26%, 16%, 58%, and 35%, resulting in an overall cage **C1** formation yield of 0.8% (Figure 2) [26]. This low yield highlights the low efficiency of cage preparation using irreversible bonds.

**Figure 3:**
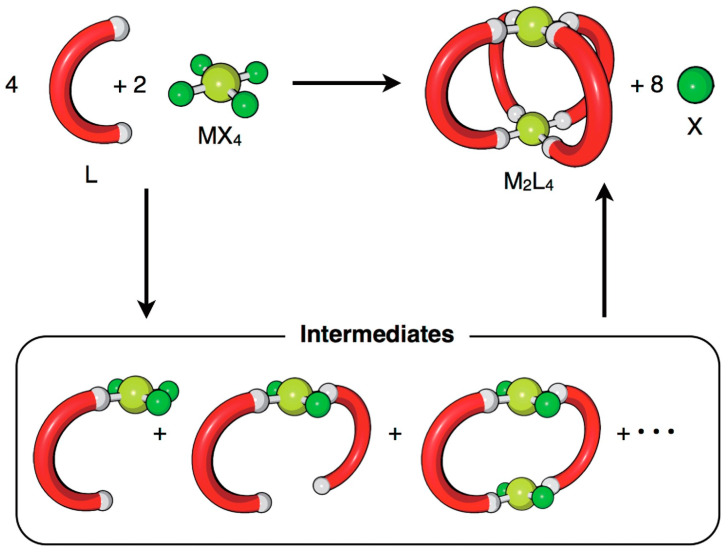
The self-assembly of an M_2_L_4_ cage from metal ion sources (MX_4_) and ditopic ligands (L). X indicates the leaving ligand, which is not a component of the final self-assembly. Reproduced with permission from reference [27]. Copyright 2018 The Chemical Society of Japan.

In contrast to the inefficiency of cage preparation using irreversible bonds, the self-assembly of cages under thermodynamic control through reversible bonds is very effective. Hiraoka and his team developed different methodologies based on qualitative and quantitative methods to investigate reversible cage formation [27]. Under such thermodynamic control conditions, the cage structures are obtained through multiple assembly pathways. This means that the molecular self-assembly proceeds through multiple reaction intermediates produced during the self-assembly process, making it difficult to detect them due to the low concentration. The method developed by Hiraoka considers intermediates as an average composition that allows monitoring of the self-assembly process through QASAP (quantitative analysis of self-assembly process) and NASAP (numerical analysis of self-assembly process) (Figure 3). These methods allowed them to determine: rate-limiting steps [28], effects of anions as templates [29], and the existence of multiple reaction pathways [30], among other parameters. 

This detailed information on cage self-assembly provides essential insights into how cages are built up, and, therefore, how to design appropriate building blocks and select synthetic conditions to obtain the target molecular cage.

### 4.1. Molecular Modelling

Another crucial aspect in the field of molecular cages is to design cavities with a specific size and shape to meet the requirements of the intended application. Experimental work has enabled access to many cage structures, providing information on key factors such as ligand/metal properties and cage formation experimental conditions. This information has been essential for developing methods for the computational design of molecular cages. The design of cages using chemical intuition is challenging, often relying on trial and error. For this reason, structure prediction using computational tools becomes important. This allows for the selection of suitable building blocks, in terms of geometry and preorganization, that will “match” the cage geometry. In this way, the success of obtaining the target cage is maximized, avoiding exploratory trial-and-error cage synthesis attempts. The tools developed in the literature allow finding out the suitability of building blocks to yield cages, predicting the most stable cage’s topology and catalytic properties as well as the volume of the cage’s cavity. The developed methods are suitable for both metal–organic cages and purely organic cages [31,32,33,34,35].

### 4.2. Strategies to Obtain Water-Soluble Molecular Cages

**Figure 4:**
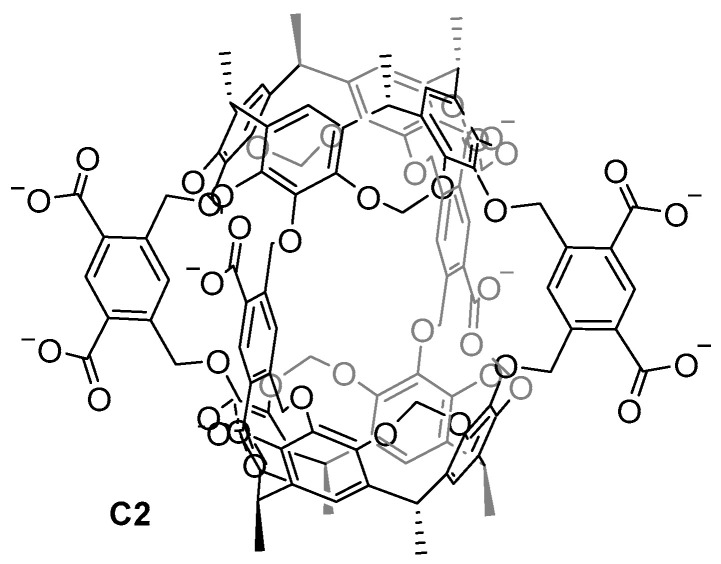
The first water-soluble hemicarcerand reported by Cram in 1997 [37].

Achieving water-soluble cage structures is crucial for their use in biological applications. For this, the use of water-solubilizing groups or cages with charges in the cage frameworks are essential factors [3,4,36]. Cram and his team prepared the first water-soluble hemicarcerand **C2** back in 1997. To overcome the hydrophobic nature of the hemicarcerands, which hinders them from being soluble in water, the authors incorporated eight carboxylate groups as water-solubilizing groups (Figure 4) [37]. This work highlights the importance of introducing charges to a hydrophobic molecule to achieve water solubility. However, the carboxylate groups have a pH dependency, becoming protonated at a low pH, which reduces the water solubility of the cage.

**Figure 5:**
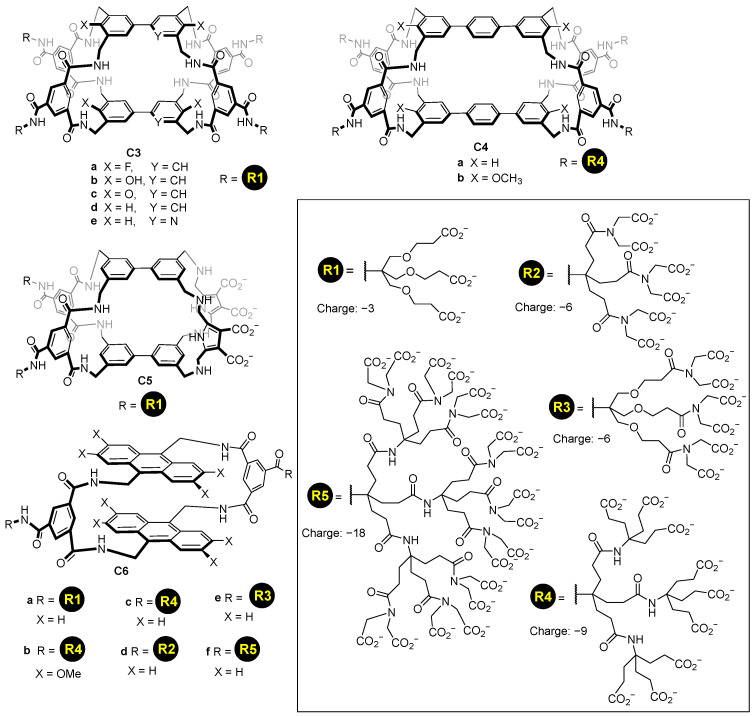
Water soluble cages featuring dendrimeric water solubilizing groups [38,39,40].

Using a similar concept, it is possible to functionalize molecular cages using, for instance, dendrimers to add multiple carboxylate groups. This enables increasing the charge introduced to the host, using different generations of dendrimers, and hence, allowing them to dissolve in water cages with a high hydrophobic nature. Davis and his team used this strategy to prepare a myriad of synthetic lectins **C3**–**C6** featuring dendrimeric carboxylate groups introducing from −3 to −18 charges per dendrimer (Figure 5) [38,39,40]. In these structures, the carboxylate groups are placed in an aromatic ring pointing outwards from the cage’s cavity.

**Figure 6:**
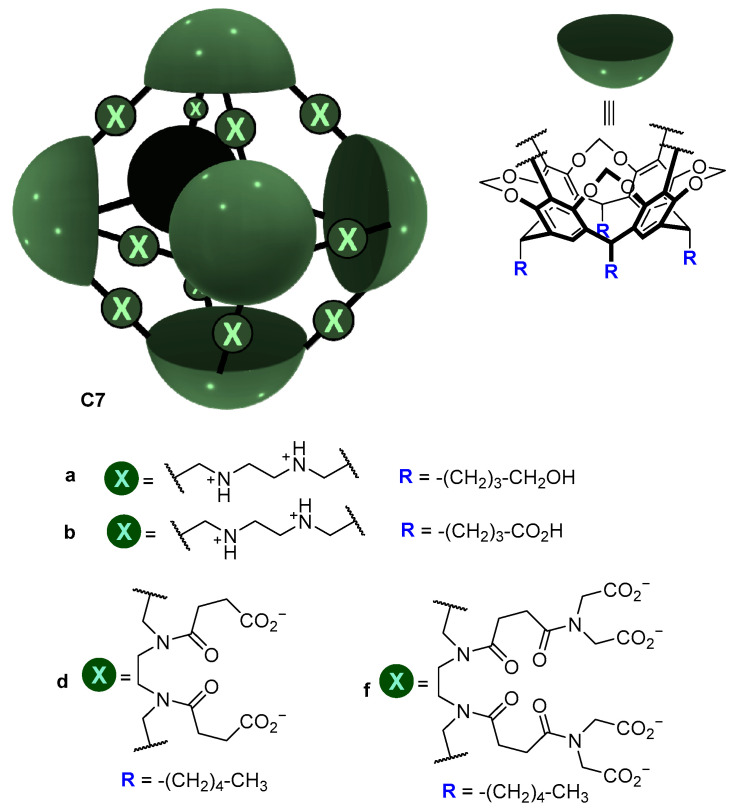
Water soluble cages and water solubilizing groups [41].

A similar strategy was used by Warmuth and co-workers, who prepared the water-soluble octahedral cage **C7** using two different strategies (Figure 6). Cage **C7** has amino groups in the cage framework that upon protonation become positively charged ammonium groups that enhance water solubility by the water solubilizing effect of the positive charges (ammonium charges are pH-dependent; they are charged at acidic pH and neutral at basic pH). To make the cage framework soluble at basic pH, the same authors introduced carboxylic groups [41]. 

**Figure 7:**
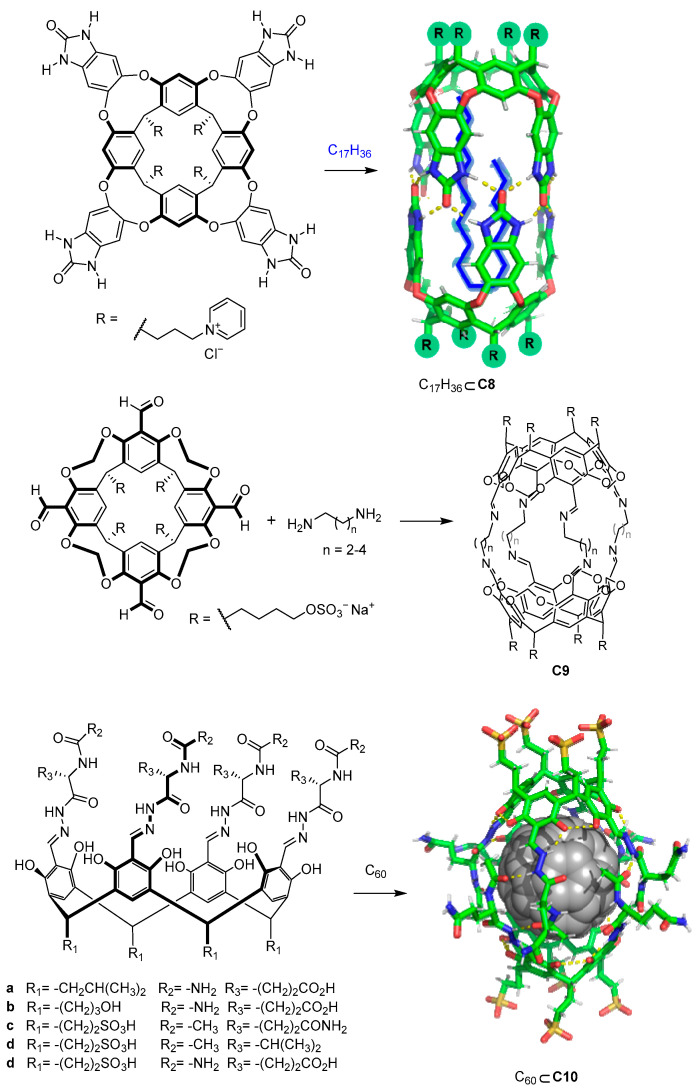
Water-soluble cavitands and molecular cages. [42,43,44].

In addition to carboxylate or amino moieties, other water-solubilizing groups such as pH-independent charged groups enable the design of water-soluble cages regardless of the pH (Figure 7). As an example, Rebek and coworkers used positively charged pyridinium moieties that conferred solubility to the cage. The reported cages were formed by the dimerization of water-soluble pyridinium cavitands around hydrophobic guests to obtain cage **C8** [42,43]. The use of sulfate or sulfonic groups, which also have a pH-independent negative charge, also allows water solubility to be obtained. For instance, Warmuth and co-workers prepared cage **C9** by incorporating R-OSO_3_^−^ water-solubilizing groups [44], whereas Szumna and co-workers prepared peptide-based water-soluble cavitands featuring sulfonic groups (R-SO_3_^−^). In the latter case, the authors observed that the cavitands self-assembled into cage **C10** in the presence of C_60_. This encapsulation process of one molecule of fullerene C_60_ is driven by the combination of C_60_–cage hydrophobic interactions and complementary hydrogen bonding between the peptide chains of the cavitands [45].

**Figure 8:**
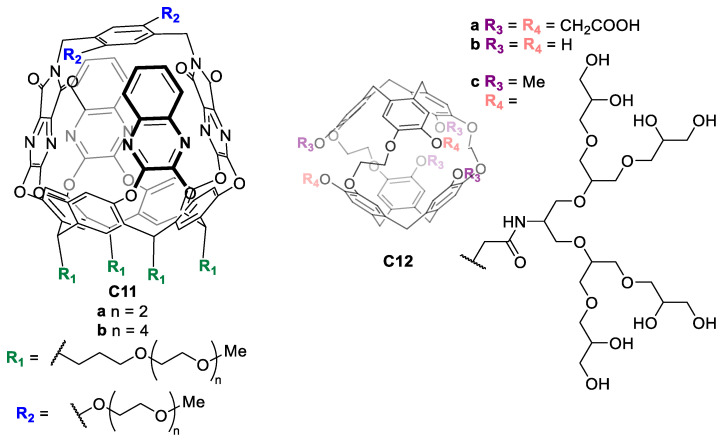
Water-soluble molecular baskets functionalized with PEG chains (**left**) and water-soluble cryptophanes functionalized with non-ionic polyglycerol dendrons (**right**) [46,47].

Other water-solubilizing groups are polyethylene glycol (PEG) or hydrophilic neutral oligomers, which can be used to enhance the water solubility of the cage. For instance, Diederich and coworkers synthesized molecular baskets **C11** based on a resorcin [4] arene cavitand functionalized with long PEG chains to achieve water solubility (Figure 8) [46]. In another example, Rousseau and coworkers prepared cryptophanes **C12** functionalized with non-ionic polyglycerol dendrons, enabling water solubility as well as biocompatibility, as these groups reduce non-specific binding to biological targets (Figure 8) [47].

**Figure 9:**
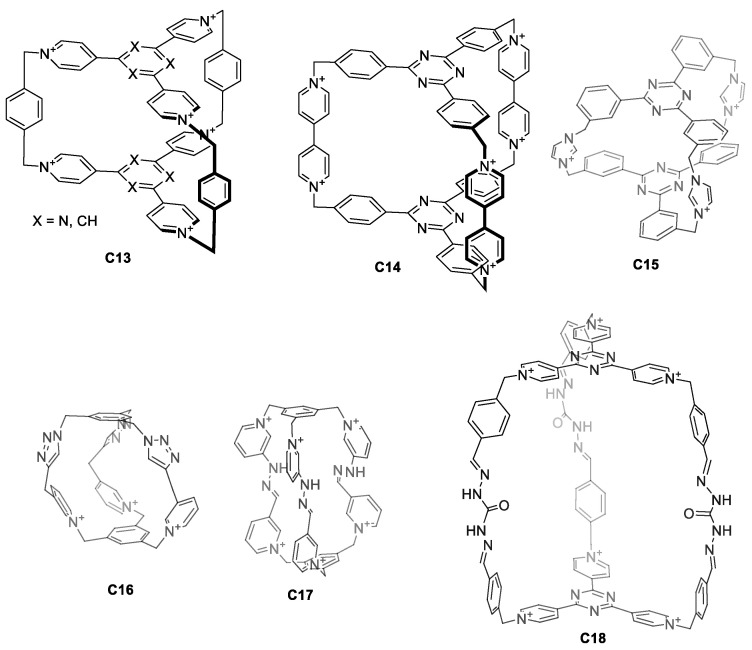
Water-soluble cages containing charged groups in the cage framework [36,48,49,50,53].

Water solubility can also be achieved by designing charged cages. The main advantage of introducing charges within the cage itself is that it simplifies the design. This eliminates the synthetic efforts to introduce external water-solubilizing components. For this, pyridinium groups have been extensively used for their synthetic simplicity as they can be obtained by a direct reaction between a pyridine moiety and an alkyl bromide. Pyridinium groups have a permanent charge that is independent of the pH. Using this strategy, Stoddart and co-workers prepared the hexacationic cages **C13** and **C14,** which contained six pyridinium rings fused with two central triazines, bridged by three *p*-xylene or 4,4′-bipyridine units (Figure 9) [48,49]. Whereas the PF_6_^−^ salts of cages **C13** and **C14** are soluble in organic solvents, the corresponding Cl^−^ salts are soluble in water, highlighting the role of the cage counteranion on solubility. A similar cage structure **C15**, with Br^−^ counter anions, was prepared by Mukherjee and his team. Despite the fact that **C15** has a tricationic charge, the cage is soluble in water. The ^1^H NMR spectra of cage **C15** in water shows broad peaks, suggesting the presence of multiple isomers. In fact, VT-NMR shows that heating the sample results in peak sharpening [50]. In contrast to the observed broad peaks, reported cages **C16**, **C17,** and **C18** display sharp ^1^H NMR peaks in water (Figure 9). The tricationic cage **C16** is water soluble with the PF_6_^−^, Cl^−^, and NO_3_^−^ counteranions [51]. In contrast, the hexacationic-charged cage **C17** with PF_6_^−^ counteranions is not soluble in water. When the counteranions of **C17** are replaced with more hydrophilic anions such as Br^−^, Cl^−^, and NO_3_^−^ the resulting cage is water soluble [52]. The cage **C18** with CF_3_CO_2_^−^ or Cl^−^ counteranions (the authors indicated that the counteranion could be either CF_3_CO_2_^−^ or Cl^−^) is also soluble in water [53].

**Figure 10:**
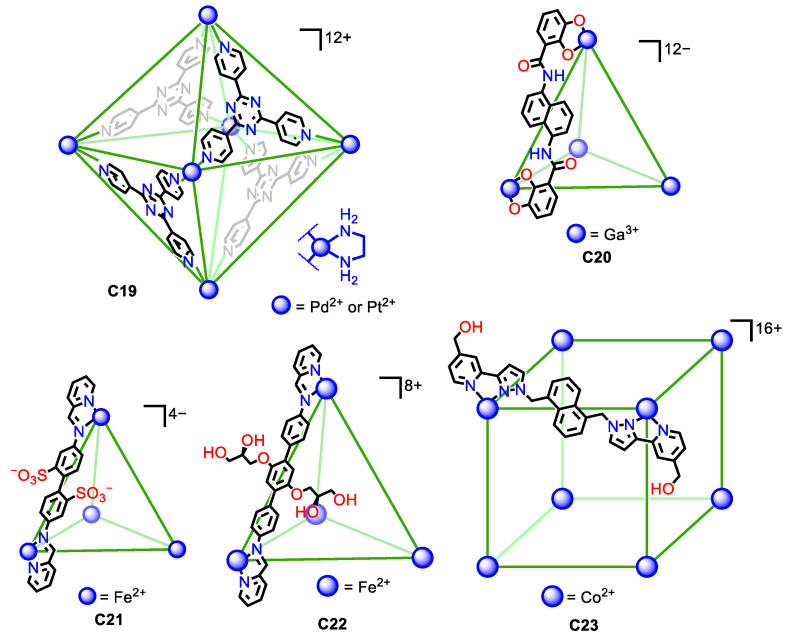
Representative examples of water soluble metal–organic cages with a high overall cage charge in the cage framework and with water solubilizing groups [54,55,56,57,58,59,60].

Water solubility in metal–organic cages can be achieved by a similar strategy. This consists of using the inherent charge of the metals that form part of the cage framework. The high number of metals that the cage structure can contain, combined with the metal charge, usually results in highly charged cages that, in most cases, provide the cage structure with sufficient water solubility. However, in some cases, the ligands in the metal-containing cages have high hydrophobicity, and it is necessary to add solubilizing groups, either charged groups or neutral groups with hydroxyl groups, that increase the water solubility of the cage assembly. We describe below representative examples of water-soluble metal–organic cages that highlight these aspects (Figure 10). 

In some cases, the high charge of the cage structure is sufficient to achieve water solubility. Fujita and his team developed a seminal example. They prepared the water-soluble octahedron **C19**, featuring either Pd(II) or Pt(II) metals. The six metals in the corners of the octahedron gave an overall +12 charge that gave water solubility to the cage assembly [54,55]. Similarly, the tetrahedral Ga_4_L_6_ **C20** cage developed by Raymond, Bergman, Toste, and co-workers had a −12-overall charge that resulted in water solubility of the assembly [56].

In other cases, just the charge of the cage assembly is not sufficient as the hydrophobic nature of the ligands hinders water solubility. To overcome this, it is necessary to increase the hydrophilicity of the ligands with polar groups. Nitschke et al. introduced the water-soluble Fe^II^_4_L_6_ **C21** tetrahedron containing sulfonate groups to give solubility to the cage assembly. The cage had an overall −4 charge and contained four Fe(II) metal centers on the tetrahedron and the six ligands with two sulfonic groups. This gave a total of 12 sulfonic groups per cage that were pointing towards the exterior, contributing to the water solubility of the assembly [57,58]. A similar situation was observed by Nitschke and his team for a similarly shaped tetrahedral molecular cage (**C22**), in this case built from terphenylene instead of biphenyl moieties. The cage had an +8-overall charge, formed by six terphenylene ligands and four Fe(II) atoms. To achieve water solubility, it was necessary to functionalize the ligand with glyceryl groups, as analogous terphenylene ligands without these groups result in a cage insoluble in water [59]. Similarly, Ward et al. prepared the water-soluble molecular cage **C23** containing eight Co(II) metal atoms at the vertices of a cube and 12 ligands. In this structure, the hydroxymethyl groups were key to achieving water solubility, as the analogous cage without these groups resulted in an insoluble cube [60]. All these examples highlight the importance, not only of the cage’s overall charge but also of the hydrophilic/hydrophobic balance of the ligands.

## 5. Biological Applications

Molecular cages exhibit promising properties for biomedical applications. In fact, the field is rapidly advancing, with progress in many therapeutic contexts. However, the biomedical applications of molecular cages are still in their infancy [61,62,63,64,65]. In the context of drug delivery, molecular cages can play a pivotal role as protective systems from the surrounding media, increasing drug stability [66,67].

In this section, we have selected some relevant examples that showcase the diverse applications of molecular cages in a biological context, mainly in cancer therapy. We have specifically chosen representative examples that demonstrate the promising potential of molecular cages in biomedical applications.

### 5.1. Cancer Therapy

**Figure 11:**
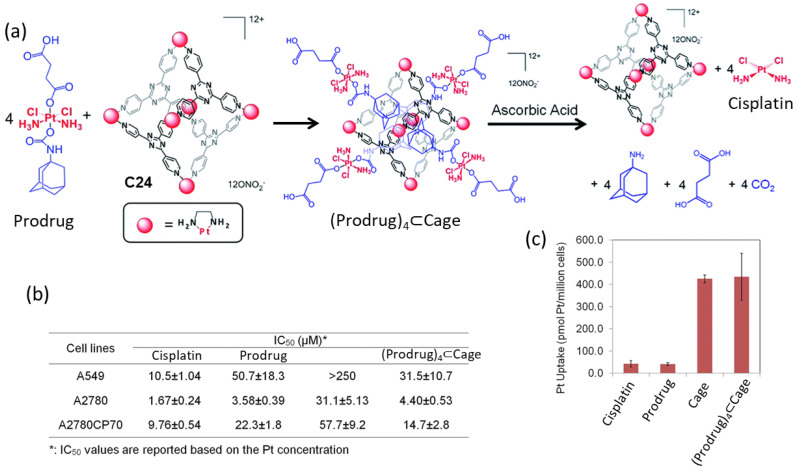
Anticancer activity of the host–guest complex assembled from the Pt(IV) prodrug and the Pt(II) cage. (**a**) Schematic representation of guest encapsulation and release by reduction with ascorbic acid. (**b**) Cytotoxicity profiles against A549 (lung cancer), A2780 (ovarian cancer), and A2780CP70 (ovarian cancer resistant to cisplatin) cell lines. (**c**) Cellular uptake in A2780CP70 cells ([Pt] = 30 μM, 4 h, 37 °C, 5% CO_2_). Adapted with permission from reference [68] with the Creative Commons CC BY license http://creativecommons.org/licenses/by/3.0/ (accessed on 3 April 2024). Copyright 2015, the authors of the original publication.

Lippard and coworkers used a Pt(II) metal–organic molecular cage **C24** to encapsulate a Pt(IV) prodrugs for anticancer drug delivery (Figure 11). The formation of the host–guest complex was achieved by mixing the Pt(IV) prodrug and the cage **C24** in a 4:1 ratio. This resulted in an improvement of the solubility of the Pt(IV) prodrug, from a low solubility in water to becoming soluble. Once the supramolecular complex (Prodrug)_4_⊂**C24** was formed, cisplatin was released in the presence of ascorbic acid. This allowed for the recovery of the anticancer activity of cisplatin. The anticancer efficacy of the system was evaluated across various human cancer cell lines, including A549 (lung carcinoma), A2780 (ovarian carcinoma), and A2780CP70 (cisplatin-resistant ovarian carcinoma), and the IC_50_ values are summarized in Figure 11. The Pt(II) cage displayed low toxicity and high cellular uptake. The supramolecular complex (Prodrug)_4_⊂**C24** displayed toxicity at micromolar concentrations against all tested cancer cell lines, akin to cisplatin. Notably, in A2780CP70 cells, Prodrug)_4_⊂**C24** exhibited enhanced cytotoxicity (IC_50_ = 14.7 μM) compared to the prodrug and **C24** (IC_50_ = 22.3 μM and 57.7 μM, respectively), underscoring the benefits of the host–guest complex. The increased cytotoxicity of (Prodrug)_4_⊂**C24** was attributed to the substantial cellular uptake of the cationic cage, which was 10-fold greater than for cisplatin or the prodrug in A2780CP70 cells. Overall, the encapsulation of the prodrug significantly enhanced its cytotoxicity by the increased cellular uptake facilitated by the cage [68]. This strategy was used by Zheng and his team, who used a fluorescein-conjugated Pt(IV) prodrug of cisplatin that resulted in the slow release of cisplatin in vitro [69].

**Figure 12:**
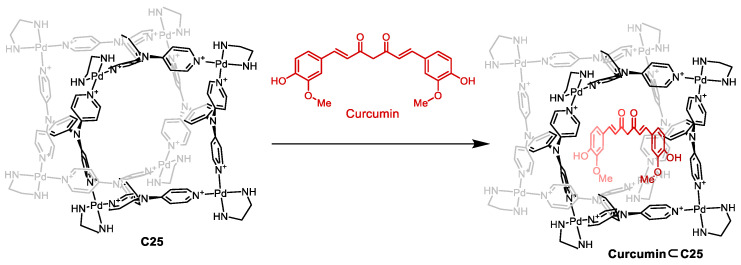
Encapsulation of curcumin in cage **C25**. Adapted with permission from Ref. [70]. Copyright 2017 American Chemical Society.

Mukherjee and co-workers prepared the [Pd_8_L_4_]^16+^ cage **C25** that featured a high charge which, in combination with nitrate counteranions, resulted in a water-soluble cage (Figure 12). The cage had a hydrophobic cavity, surrounded by phenyl and pyridine groups, which were ideal for encapsulating hydrophobic guests like curcumin. Encapsulation of curcumin resulted in enhanced photostability as the cage’s aromatic walls absorbed a high proportion of incident photons, shielding the guest against photodegradation. Additionally, the encapsulation of curcumin enhanced water solubility and cellular uptake compared to free curcumin. This resulted in free curcumin being inactive toward cancer cells. However, when curcumin was encapsulated in cage **C25**, an IC_50_ value of 14 μM was observed. This activity is similar to free curcumin in DMSO, which has an IC_50_ value of 10 μM [70].

**Figure 13:**
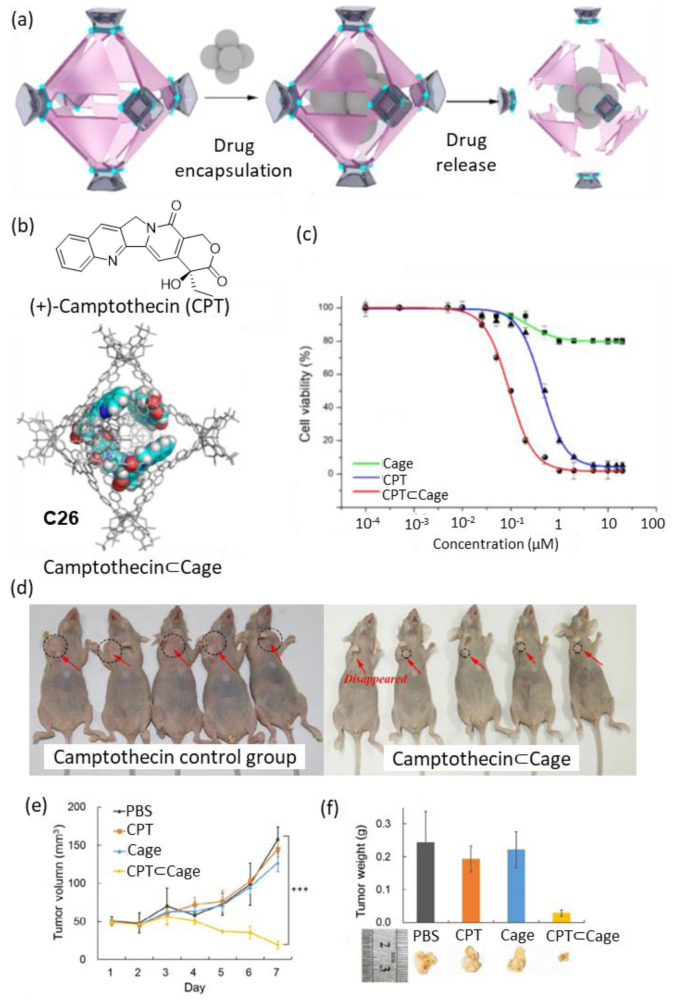
(**a**) Schematic representation of the encapsulation of Camptothecin followed by drug release by cage disassembly. (**b**) Chemical structure of Camptothecin and encapsulation of four molecules of camptothecin in the cage cavity. (**c**) Representative example of in vitro cell viability experiments showing IC_50_ of HeLa cells. (**d**) Photographs of tumors after seven days from tumor inhibition of Camptothecin⊂**C26** (concentration: 21.6 nM based on Camptothecin, HepG2 cell xenograft model in BALB/c nude mice). (**e**) Evolution of tumor volumes during the 7-day treatment. (**f**) Tumor weights and pictures of the different groups on day 7. (*** *p* < 0.005; Student’s two-tailed t-test). Adapted with permission from reference [71]. Copyright 2021, Springer.

Zhou and coworkers developed a metal–organic cage that decomposed gradually, releasing the encapsulated drug at a constant rate (Figure 13). The cage showed complete decomposition at physiological pH (i.e., pH = 7.4) in seven days, with an acceleration of the decomposition kinetics when the acidity of the media was increased to pH = 6.5. The cage encapsulated four molecules of the anticancer drug Camptothecin (Figure 13b). Cell viability in vitro was tested in four human cancer cell lines: HeLa, SKOV3 (ovarian carcinoma), H1299 (lung cancer), and HepG2 (liver hepatocellular carcinoma). Concentrations of free cage around 1 µM resulted in cell viabilities of approximately 90%, indicating the low toxicity of the cage. When Camptothecin was encapsulated in cage **C26** a value of IC_50_ = 0.09 μM was found, which contrasted with the IC_50_ = 0.68 μM of free Camptothecin (Figure 13c). The authors also tested the anticancer activity of Camptothecin⊂Cage in vivo in a xenograft tumor mouse model of HepG2 cells in BALB/c nude mice. For this, an intravenous injection of Camptothecin⊂Cage (daily administration of 200 µL with concentration 21.6 nM for 7 days) and the corresponding controls were carried out. It was observed that the Camptothecin⊂Cage had a significant effect on the reduction of the tumor volume, demonstrating the efficiency of the cage as a sustained drug release agent (Figure 13d–f) [71,72]. 

**Figure 14:**
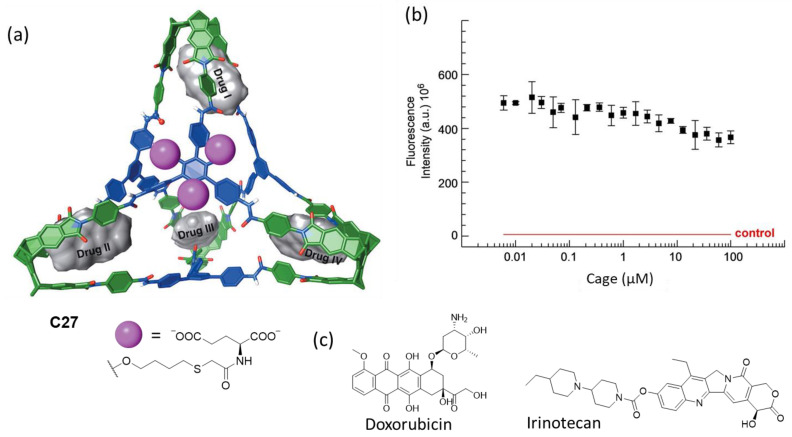
(**a**) Chemical structure of the water-soluble molecular cage with the four hydrophobic binding pockets and chemical structures of the anticancer drugs irinotecan and doxorubicin. (**b**) Cell viability assay displaying the fluorescence intensity of HCT 116 cell line in a medium containing resazurin dye as a function of cage concentration after 48 h of incubation. The result of a control experiment without cells (death control) is shown in red. (**c**) Anticancer guest molecules that the cage encapsulates. Adapted with permission from reference [73]. Copyright 2023, Wiley.

Badjić and his team prepared the water-soluble molecular cage **C27** featuring carboxylic groups as water-solubilizing elements for the efficient encapsulation of anticancer drugs as nanoantidotes for the rapid sequestration of toxic molecules (Figure 14). The amphiphilic nature of the cage molecules resulted in aggregation, with a critical aggregation concentration of less than 1 μM, resulting in spherical nanoparticle aggregates with a diameter of roughly 250 nm. The molecular cage encapsulated the anticancer drugs irinotecan and doxorubicin, with four encapsulated drug molecules in the hydrophobic pockets of the cage. The cage had a negligible effect on the viability of the human colon carcinoma cell line HCT 116 at 100 μM concentration [73].

Zheng and coworkers prepared the Pt_6_L_4_ octahedral cage **C28** as an anticancer agent containing the chelating 1,10-phenanthroline moiety that has potential activity to generate DNA damage. The cytotoxicity profiles of cage **C28** against different human cancer cell lines including ovarian cancer A2780, cisplatin-resistant ovarian A2780cis, ovarian cancer SKOV-3, lung cancer A549, and triple-negative breast cancer MDA-MB-231, were tested. Whereas cage **C28** exhibited IC_50_ values comparable to cisplatin against A2780, A549, and MDA-MB-231 cell lines, the cytotoxicity against the cisplatin-resistant A2780cis cell line (IC_50_ = 4.5 μM) was enhanced in comparison to cisplatin (IC_50_ = 11.5 μM). For this cancer cell line, immunoblotting analysis showed an increase in the phosphorylated H2AX (γH2AX) DNA-damage biomarker, confirming the action mode of the cage.

**Figure 15:**
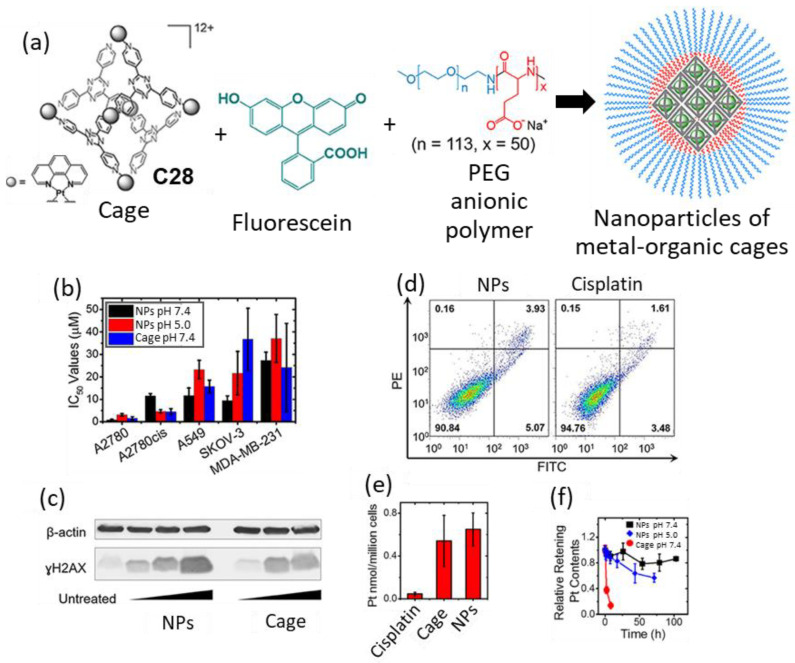
(**a**) Graphical representation of the formation of nanoparticles of metal–organic cages with fluorescein forming a 1:1 host–guest complex, MPEG5k-PGA50 anionic block copolymer enables the nanoprecipitation of this complex into nMOC, (**b**) IC_50_ values of cisplatin, cage and NPs on different human cancer cell lines. (**c**) Immunoblotting analysis of γH2AX in A2780cis cell line. (**d**) Annexin V apoptosis assay using A2780cis cells treated with NPs (left) and cisplatin (right). (**e**) Cellular uptake profiles of the different Pt compounds in A2780cis ovarian cancer cells analyzed by using GFAAS. (**f**) Drug release profile of NPs and cage in PBS (pH = 7.4) or acetate buffer (pH = 5.0) at R.T. Adapted with permission from reference [74] with the Creative Commons CC BY license http://creativecommons.org/licenses/by/4.0/ (accessed on 3 April 2024). Copyright 2019, the authors of the original publication.

Based on these good results, further development was performed using a formulation to improve the limited solubility of the cage in cell culture media (<20 μM) due to its hydrophobic nature. This was achieved using the MPEG_5k_-PGA_50_ anionic block copolymer and fluorescein that induces nanoprecipitation driven by interactions between the three components (Figure 15). The nanoprecipitation process consisted of fluorescein encapsulation to form a 1:1 host–guest complex that formed the core of the nanoparticle that was surrounded by MPEG_5k_-PGA_50_ polymer chains. The resulting nanoparticles had a size of 100 nm and enhanced the solubility of cage **C28,** which could be dissolved to values up to 0.4 mM in aqueous solution and cell culture media. Additionally, the nanoparticles showed a slow release of Pt contents in PBS (pH = 7.4); 20% of Pt contents were released after 100 h, as determined using dialysis. Additional studies demonstrated that both free cage **C28** and nanoparticles can efficiently enter cancer cells. Both showed a 10-fold cellular uptake increase with regard to cisplatin in A2780cis cells. Cytotoxicity studies of the nanoparticles against the 2780, A2780cis, A549, SKOV-3, and MDA-MB-231 cancer cell lines showed that nanoparticles have IC_50_ values comparable to those of the free cage. Overall, the nanoparticles are a promising system for delivering cytotoxic metal–organic cages [74].

**Figure 16:**
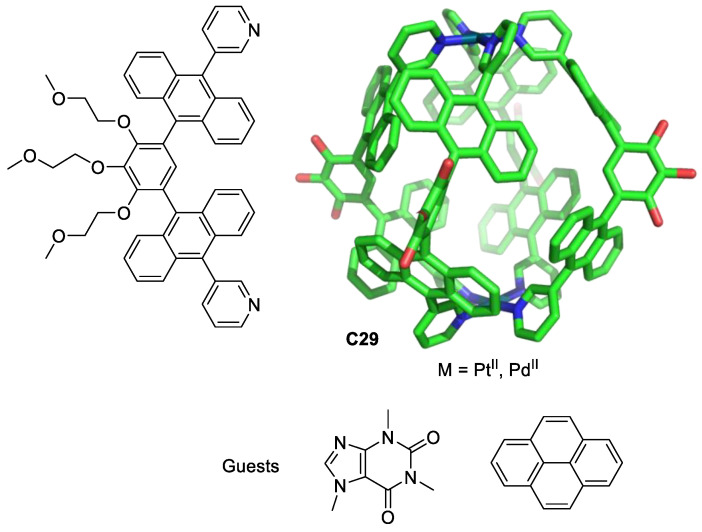
Chemical structure of the ligand and the corresponding cage **C29** (Pd^II^ or Pt^II^, hydrogen atoms and PEG groups omitted for clarity), and pyrene and caffeine guests [75].

Yoshizawa, Ahmedova, and their team used M_2_L_4_ metal–organic cages as anticancer agents against different human cancer cells (HT-29, T-24, HL-60 and their resistant counterparts HL-60/Dox, and HL-60/CDDP). The cages **C29** were formed by Pt(II) or Pd(II) atoms linked by four ligands. These cages have a hydrophobic cavity surrounded by aromatic groups that encapsulate neutral molecules through hydrophobic and/or π-stacking interactions in aqueous solutions (Figure 16). The cages **C29** form 1:2 host–guest complexes with pyrene and caffeine. The anticancer activity is modulated by guest encapsulation (Table 1). Pyrene encapsulation reduces cytotoxicity against the less chemosensitive cells T-24 and HT-29, and caffeine encapsulation increases cytotoxicity against HL-60, HL-60/CDDP, and HT-29 compared with that of cisplatin [75,76].

### 5.2. Other Biological Applications

**Figure 17:**
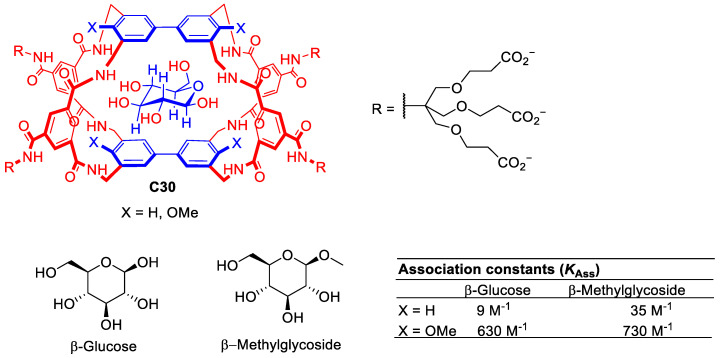
Chemical structure of cage **C30** with complementary apolar groups are shown in blue, polar units in red. The structure showing the expected positioning of the guest [78].

Davies and his team developed numerous synthetic lectins able to recognize multiple carbohydrate molecules. The design of carbohydrate hosts is challenging as the carbohydrate guest must reject water molecules from the cavity to establish new host–guest interactions which are very similar, i.e., both water and carbohydrate have hydroxyl groups. To approach this challenge, the authors designed cages with the correct size and shape to target carbohydrates (Figure 17). This design considered appropriate host and guest matches for polar regions (e.g., H-bond donor for H-acceptor, etc.) and hydrophobic surfaces [77]. Representative examples of these systems are described in Figure 5. For example, molecular cage **C30** shows promise for binding carbohydrate substrates due to its tunable binding properties achieved by modifying the substituents of the biphenyl units. While glucose binding remains moderate using **C30**, the methoxy substituents enhance selectivity for β-methylglucoside [78].

**Figure 18:**
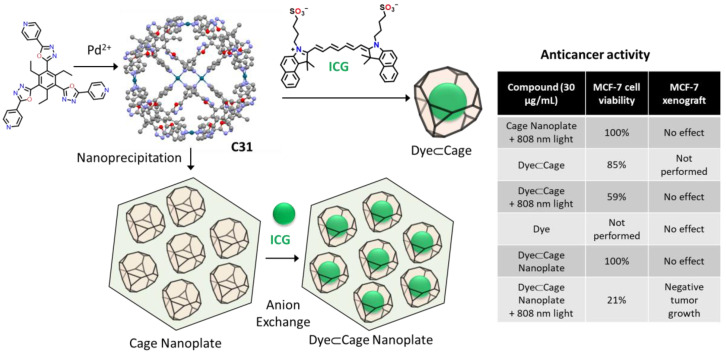
Nanoprecipitation–anion-exchange with indocyanine green of a Pd_6_L_8_ metal–organic cage with photodynamic antitumor therapy [79].

Dong, Li, and coworkers used the Pd_6_L_8_ metal–organic cage **C31** for photodynamic antitumor therapy (Figure 18). Using nanoprecipitation–anion-exchange the authors prepared nanoplates formed by cage molecules containing an average of 6.6 molecules of indocyanine green per cage. A significant, red-shift color change was observed during anion exchange, implying a strong indocyanine green–cage interaction. The obtained nanoplates were stable under physiological conditions, allowing their use in photodynamic antitumor therapy experiments in vitro (MCF-7 human breast adenocarcinoma cell line) and in vivo (MCF-7 xenograft in nude mice). In both experiments, the maximum efficiency was obtained for the cage-dye nanoplate irradiated with an 808 nm laser. In these conditions, the system showed enhanced near-infrared triggered ^1^O_2_ generation, high cellular uptake, and selective lysosome-targeting [79].

**Figure 19:**
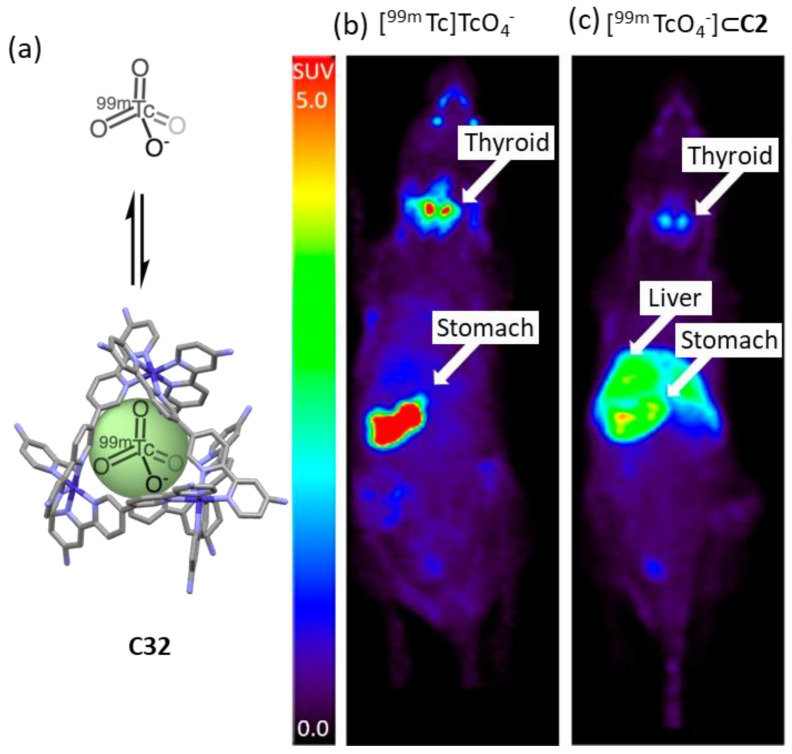
Comparison of [^99m^Tc]TcO_4_^−^ uptake in naïve mice vs [^99m^Tc][TcO_4_⊂**C32**]^11+^. Encapsulation results in reduced thyroid and stomach uptake, and increased liver uptake. (**a**) Encapsulation of [^99m^Tc]TcO_4_^−^ in **C32**. (**b**) Free [^99m^Tc]TcO_4_^−^ shows preferential accumulation in thyroid. (**c**) Complex ^99m^Tc][TcO_4_⊂**C32**]^11+^ shows preferential accumulation in the liver. Compounds were injected into a naïve anesthetized animal followed by SPECT acquisition. Images are maximum intensity coronal projections [80]. Adapted with permission from reference [80] with the Creative Commons CC BY license http://creativecommons.org/licenses/by/4.0/ (accessed on 3 April 2024). Copyright 2018, the authors of the original publication.

Lusby and his team developed a Co^III^_4_L_6_ cage to encapsulate the γ-emitting radiochemical [^99m^Tc]TcO_4_^−^, the most widely used precursor in clinical nuclear diagnostic imaging (Figure 19). The system was stable in biological media, resulting in a system compatible with in vivo administration. While the administration of free [^99m^Tc]TcO_4_^−^ to naïve mice followed by in vivo SPECT imaging showed the expected preferential uptake in the thyroid ([^99m^Tc]TcO_4_^−^ is used clinically to measure thyroid function), the administration of [^99m^Tc][TcO_4_⊂**C32**]^11+^ showed a significant uptake in the liver. These results highlight that the encapsulation changes the biodistribution, reducing thyroid and stomach uptake while increasing liver uptake [80].

## 6. Conclusions and Future Directions

The field of molecular cages shows growing potential for biological applications, with remarkable examples reported in recent years. One of the main challenges for this is achieving the required water solubility while overcoming the inherent hydrophobic nature of molecular cages, due to the organic building block components. Water solubility can be achieved using two strategies: (a) adding water-solubilizing groups that contain negative or positive charges or polyethylene glycol chains; and (b) adding charges to the cage structure itself to obtain a highly charged molecular cage. These strategies allow preparing water-soluble molecular cages. Biological applications of cages include anticancer activity of the cage itself, anticancer drug delivery, photodynamic therapy, or molecular recognition of biological molecules. For in vivo applications, the metabolic behaviors of molecular cages and cage-based nanoparticles are an important aspect to be considered in the future directions of the field. A rising number of applications are expected in the coming years by the fine-tuning of cage properties that will allow reaching different biological applications that are not possible to achieve with other molecular systems.

## Figures and Tables

**Table 1:** Cytotoxicity (IC_50_ values, 72 h of treatment) of (pyrene or caffeine)_2_**⊂C29** and free cage **C29** against HL-60, HL-60/Dox, T-24, and HT-29, compared with free pyrene, caffeine, ligand, and cisplatin [75].

Compound	HL-60	HL-60/Dox	HT-29	T-24
(pyrene)_2_**⊂C29**-Pt	22.8	21.5	109.4	>100
(caffeine)_2_**⊂C29**-Pt	2.6	5.0	18.2	>100
**C29**-Pt	5.0	1.0	0.9	5.3
(pyrene)_2_**⊂C29**-Pd	2.5	8.4	69.3	>100
(caffeine)_2_**⊂C29**-Pd	0.7	4.7	23.2	>100
**C29**-Pd	1.6	1.1	17.5	37.4
Ligand, pyrene, caffeine	>100	>100	>100	>100
Cisplatin	9.3	32.9	36.6	13.5

## References

[1] Lewis J.E.M. (2023). Developing Sophisticated Microenvironments in Metal-organic Cages. Trends Chem..

[2] Martí-Centelles V., Duarte F., Lusby P.J. (2019). Host-Guest Chemistry of Self-Assembled Hemi-Cage Systems: The Dramatic Effect of Lost Pre-Organization. Isr. J. Chem..

[3] Montà-González G., Sancenón F., Martínez-Máñez R., Martí-Centelles V. (2022). Purely Covalent Molecular Cages and Containers for Guest Encapsulation. Chem. Rev..

[4] Percástegui E.G., Ronson T.K., Nitschke J.R. (2020). Design and Applications of Water-soluble Coordination Cages. Chem. Rev..

[5] Takezawa H., Fujita M. (2021). Molecular Confinement Effects by Self-assembled Coordination Cages. Bull. Chem. Soc. Jpn..

[6] Martí-Centelles V., Lawrence A.L., Lusby P.J. (2018). High Activity and Efficient Turnover by a Simple, Self-Assembled Artificial “Diels-Alderase”. J. Am. Chem. Soc..

[7] Yu Y., Yang J.M., Rebek J. (2020). Molecules in Confined Spaces: Reactivities and Possibilities in Cavitands. Chem.

[8] Pappalardo A., Puglisi R., Sfrazzetto G.T. (2019). Catalysis inside Supramolecular Capsules: Recent Developments. Catalysts.

[9] Chen S., Chen L.-J. (2022). Metal–organic Cages: Applications in Organic Reactions. Chemistry.

[10] Mal P., Breiner B., Rissanen K., Nitschke J.R. (2009). White Phosphorus is Air-Stable within a Self-Assembled Tetrahedral Capsule. Science.

[11] Galan A., Ballester P. (2016). Stabilization of Reactive Species by Supramolecular Encapsulation. Chem. Soc. Rev..

[12] Zhang D., Ronson T.K., Zou Y.-Q., Nitschke J.R. (2021). Metal–organic Cages for Molecular Separations. Nat. Rev. Chem..

[13] Little M.A., Cooper A.I. (2020). The Chemistry of Porous Organic Molecular Materials. Adv. Funct. Mater..

[14] Zhang J., Xie S., Zi M., Yuan L. (2020). Recent Advances of Application of Porous Molecular Cages for Enantioselective Recognition and Separation. J. Sep. Sci..

[15] Zhang G., Mastalerz M. (2014). Organic Cage Compounds-from Shape-Persistency to Function. Chem. Soc. Rev..

[16] Liu W., Stoddart J.F. (2021). Emergent Behavior in Nanoconfined Molecular Containers. Chem.

[17] Singh M., Sharma R., Banerjee U.C. (2002). Biotechnological applications of cyclodextrins. Biotechnol. Adv..

[18] Challa R., Ahuja A., Ali J., Khar R.K. (2005). Cyclodextrins in Drug Delivery: An Updated Review. AAPS PharmSciTech.

[19] Das D., Assaf K.I., Nau W.M. (2019). Applications of Cucurbiturils in Medicinal Chemistry and Chemical Biology. Front. Chem..

[20] Martí-Centelles V., Pandey M.D., Burguete M.I., Luis S.V. (2015). Macrocyclization Reactions: The Importance of Conformational, Configurational, and Template-Induced Preorganization. Chem. Rev..

[21] Martí-Centelles V. (2022). Kinetic and thermodynamic concepts as synthetic tools in supramolecular chemistry for preparing macrocycles and molecular cages. Tetrahedron Lett..

[22] Lewis J.E.M. (2022). Molecular Engineering of Confined Space in Metal–organic Cages. Chem. Commun..

[23] Tateishi T., Yoshimura M., Tokuda S., Matsuda F., Fujita D., Furukawa S. (2022). Coordination/metal–organic cages inside out. Coord. Chem. Rev..

[24] Yang X., Ullah Z., Stoddart J.F., Yavuz C.T. (2023). Porous Organic Cages. Chem. Rev..

[25] Rowan S.J., Cantrill S.J., Cousins G.R.L., Sanders J.K.M., Stoddart J.F. (2002). Dynamic Covalent Chemistry. Angew. Chem. Int. Ed..

[26] Makeiff D.A., Sherman J.C. (2005). A Six-Bowl Carceplex That Entraps Seven Guest Molecules. J. Am. Chem. Soc..

[27] Hiraoka S. (2018). Unresolved Issues that Remain in Molecular Self-Assembly. Bull. Chem. Soc. Jpn..

[28] Kai S., Martí-Centelles V., Sakuma Y., Mashiko T., Kojima T., Nagashima U., Tachikawa M., Lusby P.J., Hiraoka S. (2018). Quantitative Analysis of Self-assembly Process of a Pd_2_L_4_ Cage Consisting of Rigid Ditopic Ligands. Chem. Eur. J..

[29] Tateishi T., Takahashi S., Okazawa A., Martí-Centelles V., Wang J., Kojima T., Lusby P.J., Sato H., Hiraoka S. (2019). Navigated Self-Assembly of a Pd_2_L_4_ Cage by Modulation of an Energy Landscape under Kinetic Control. J. Am. Chem. Soc..

[30] Tateishi T., Kojima T., Hiraoka S. (2018). Multiple Pathways in the Self-Assembly Process of a Pd_4_L_8_ Coordination Tetrahedron. Inorg. Chem..

[31] Santolini V., Miklitz M., Berardo E., Jelfs K.E. (2017). Topological Landscapes of Porous Organic Cages. Nanoscale.

[32] Piskorz T.K., Martí-Centelles V., Young T.A., Lusby P.J., Duarte F. (2022). Computational Modeling of Supramolecular Metallo-organic Cages—Challenges and Opportunities. ACS Catal..

[33] Tarzia A., Jelfs K.E. (2022). Unlocking the Computational Design of Metal–organic Cages. Chem. Comm..

[34] Greenaway R.L., Jelfs K.E. (2020). High-throughput Approaches for the Discovery of Supramolecular Organic Cages. ChemPlusChem.

[35] Martí-Centelles V., Piskorz T.K., Duarte F. (2024). Cagecavitycalc (C3): A Computational Tool for Calculating and Visualizing Cavities in Molecular Cages. ChemRxiv.

[36] Chakraborty D., Mukherjee P.S. (2022). Recent Trends in Organic Cage Synthesis: Push towards Water-soluble Organic Cages. Chem. Commun..

[37] Yoon J., Cram D.J. (1997). The First Water-Soluble Hermicarceplexes. Chem. Commun..

[38] Barwell N.P., Davis A.P. (2011). Substituent Effects in Synthetic Lectins—Exploring the Role of CH-π Interactions in Carbohydrate Recognition. J. Org. Chem..

[39] Howgego J.D., Butts C.P., Crump M.P., Davis A.P. (2013). An Accessible Bicyclic Architecture for Synthetic Lectins. Chem. Commun..

[40] Joshi G., Davis A.P. (2012). New H-Bonding Patterns in Biphenyl-Based Synthetic Lectins; Pyrrolediamine Bridges Enhance Glucose Selectivity. Org. Biomol. Chem..

[41] Liu X., Sun J., Warmuth R. (2009). Water-Soluble Octahedral Polyammonium Nanocapsules: Synthesis and Encapsulation Studies. Tetrahedron.

[42] Gavette J.V., Zhang K.D., Ajami D., Rebek J. (2014). Folded Alkyl Chains in Water-Soluble Capsules and Cavitands. Org. Biomol. Chem..

[43] Gavette J.V., Petsalakis I.D., Theodorakopoulos G., Zhang K.D., Yu Y., Rebek J. (2015). The Effects of Hexafluoroisopropanol on Guest Binding by Water-Soluble Capsule and Cavitand Hosts. Chem. Commun..

[44] Lin Z., Sun J., Efremovska B., Warmuth R. (2012). Assembly of Water-Soluble, Dynamic, Covalent Container Molecules and Their Application in the Room-Temperature Stabilization of Protoadamantene. Chem. Eur. J..

[45] Eichstaedt K., Szpotkowski K., Grajda M., Gilski M., Wosicki S., Jaskólski M., Szumna A. (2019). Self-assembly and Ordering of Peptide-based Cavitands in Water and DMSO: The Power of Hydrophobic Effects Combined with Neutral Hydrogen Bonds. Chem. Eur. J..

[46] Fankhauser D., Kolarski D., Grüning W.R., Diederich F. (2014). Resorcin[4]Arene-Based Molecular Baskets and Water-Soluble Container Molecules: Synthesis and ^1^H NMR Host-Guest Complexation Studies. Eur. J. Org. Chem..

[47] Tyagi R., Witte C., Haag R., Schröder L. (2014). Dendronized Cryptophanes as Water-Soluble Xenon Hosts for ^129^Xe Magnetic Resonance Imaging. Org. Lett..

[48] Hafezi N., Holcroft J.M., Hartlieb K.J., Dale E.J., Vermeulen N.A., Stern C.L., Sarjeant A.A., Stoddart J.F. (2015). Modulating the Binding of Polycyclic Aromatic Hydrocarbons Inside a Hexacationic Cage by Anion-π Interactions. Angew. Chem. Int. Ed..

[49] Jiao T., Cai K., Liu Z., Wu G., Shen L., Cheng C., Feng Y., Stern C.L., Stoddart J.F., Li H. (2019). Guest Recognition Enhanced by Lateral Interactions. Chem. Sci..

[50] Chakraborty D., Modak R., Howlader P., Mukherjee P.S. (2021). De Novo Approach for Synthesis of Water-Soluble Interlocked and Non-Interloked Organic Cages. Chem. Commun..

[51] Chen Y., Wu G., Chen L., Tong L., Lei Y., Shen L., Jiao T., Li H. (2020). Selective Recognition of Chloride Anion in Water. Org. Lett..

[52] Wang H., Fang S., Wu G., Lei Y., Chen Q., Wang H., Wu Y., Lin C., Hong X., Kim S.K. (2020). Constraining Homo- and Heteroanion Dimers in Ultraclose Proximity within a Self-Assembled Hexacationic Cage. J. Am. Chem. Soc..

[53] Zheng X., Zhang Y., Wu G., Liu J.R., Cao N., Wang L., Wang Y., Li X., Hong X., Yang C. (2018). Temperature-Dependent Self-Assembly of a Purely Organic Cage in Water. Chem. Commun..

[54] Fujita M., Oguro D., Miyazawa M., Oka H., Yamaguchi K., Ogura K. (1995). Self-Assembly of Ten Molecules into Nanometer-Sized Host Frameworks. Nature.

[55] Ibukuro F., Kusukawa T., Fujita M. (1998). A Thermally Switchable Molecular Lock. Guest-Templated Synthesis of a Kinetically Stable Nanosized Cage. J. Am. Chem. Soc..

[56] Hastings C.J., Pluth M.D., Bergman R.G., Raymond K.N. (2010). Enzymelike Catalysis of the Nazarov Cyclization by Supramolecular Encapsulation. J. Am. Chem. Soc..

[57] Mal P., Schultz D., Beyeh K., Rissanen K., Nitschke J.R. (2008). An Unlockable-Relockable Iron Cage by Subcomponent Self-Assembly. Angew. Chem. Int. Ed..

[58] Clegg J.K., Cremers J., Hogben A.J., Breiner B., Smulders M.M.J., Thoburn J.D., Nitschke J.R. (2013). A Stimuli Responsive System of Self-Assembled Anion-Binding Fe_4_L_6_^8+^ Cages. Chem. Sci..

[59] Bolliger J.L., Belenguer A.M., Nitschke J.R. (2013). Enantiopure Water-Soluble [Fe_4_L_6_] Cages: Host-Guest Chemistry and Catalytic Activity. Angew. Chem. Int. Ed..

[60] Whitehead M., Turega S., Stephenson A., Hunter C.A., Ward M.D. (2013). Quantification of Solvent Effects on Molecular Recognition in Polyhedral Coordination Cage Hosts. Chem. Sci..

[61] Casini A., Woods B., Wenzel M. (2017). The Promise of Self-Assembled 3D Supramolecular Coordination Complexes for Biomedical Applications. Inorg. Chem..

[62] Zhu C.-Y., Pan M., Su C.-Y. (2018). Metal-Organic Cages for Biomedical Applications. Isr. J. Chem..

[63] Dou W.-T., Yang C.-Y., Hu L.-R., Song B., Jin T., Jia P.-P., Ji X., Zheng F., Yang H.-B., Xu L. (2023). Metallacages and Covalent Cages for Biological Imaging and Therapeutics. ACS Mater. Lett..

[64] Ahmad N., Younus H.A., Chughtai A.H., Verpoort F. (2015). Metal–organic Molecular Cages: Applications of Biochemical Implications. Chem. Soc. Rev..

[65] Sun D., Feng X., Zhu X., Wang Y., Yang J. (2024). Anticancer Agents Based on Metal Organic Cages. Coord. Chem. Rev..

[66] Cruz-Nava S., De Jesús Valencia-Loza S., Percástegui E.G. (2022). Protection and Transformation of Natural Products within Aqueous Metal-organic Cages. Eur. J. Org. Chem..

[67] Tapia L., Alfonso I., Solà J. (2021). Molecular Cages for Biological Applications. Org. Biomol. Chem..

[68] Zheng Y.-R., Suntharalingam K., Johnstone T.C., Lippard S.J. (2015). Encapsulation of Pt(iv) Prodrugs Within a Pt(ii) Cage for Drug Delivery. Chem. Sci..

[69] Yue Z., Wang H., Bowers D.J., Gao M., Stilgenbauer M., Nielsen F., Shelley J.T., Zheng Y.-R. (2018). Nanoparticles of Metal–organic Cages Designed to Encapsulate Platinum-based Anticancer Agents. Dalton Trans..

[70] Bhat I.A., Jain R., Siddiqui M.M., Saini D.K., Mukherjee P.S. (2017). Water-Soluble Pd_8_L_4_ Self-assembled Molecular Barrel as an Aqueous Carrier for Hydrophobic Curcumin. Inorg. Chem..

[71] Liang Y., Fang Y., Cui Y., Zhou H. (2021). A Stable Biocompatible Porous Coordination Cage Promotes In Vivo Liver Tumor Inhibition. Nano Res..

[72] Fang Y., Lian X., Huang Y., Fu G., Xiao Z., Wang Q., Nan B., Pellois J., Zhou H. (2018). Investigating Subcellular Compartment Targeting Effect of Porous Coordination Cages for Enhancing Cancer Nanotherapy. Small.

[73] Gunawardana V.W.L., Ward C., Wang H., Holbrook J.H., Sekera E.R., Cui H., Hummon A.B., Badjić J.D. (2023). Crystalline Nanoparticles of Water-soluble Covalent Basket Cages (cbcs) for Encapsulation of Anticancer Drugs. Angew. Chem. Int. Ed..

[74] Wang H., Qiu Z., Liu H., Jayawardhana A.M.D.S., Yue Z., Daghlas H., Bowers D.J., Datta B., Zheng Y.-R. (2019). Nanoparticles of Metal-organic Cages Overcoming Drug Resistance in Ovarian Cancer. Front. Chem..

[75] Ahmedova A., Mihaylova R., Momekova D., Shestakova P., Stoykova S., Zaharieva J., Yamashina M., Momekov G., Akita M., Yoshizawa M. (2016). M_2_L_4_ Coordination Capsules with Tunable Anticancer Activity upon Guest Encapsulation. Dalton Trans..

[76] Ahmedova A., Momekova D., Yamashina M., Shestakova P., Momekov G., Akita M., Yoshizawa M. (2016). Anticancer Potencies of Pt^ii^- and Pd^ii^-linked M_2_L_4_ Coordination Capsules with Improved Selectivity. Chem. Asian J..

[77] Davis A.P. (2009). Synthetic Lectins. Org. Biomol. Chem..

[78] Barwell N.P., Crump M.P., Davis A.P. (2009). A Synthetic Lectin for Β-glucosyl. Angew. Chem. Int. Ed..

[79] Li W.-Y., Zhao C.-W., Zhang Y.-F., Guan Q., Wan J.-J., Ma J.-P., Li Y.-A., Dong Y.-B. (2021). A metal–organic cage-based nanoagent for enhanced photodynamic antitumor therapy. Chem. Commun..

[80] Burke B.P., Grantham W., Burke M.J., Nichol G.S., Roberts D., Renard I., Hargreaves R., Cawthorne C., Archibald S.J., Lusby P.J. (2018). Visualizing Kinetically Robust Co^III^_4_L_6_ Assemblies In Vivo: SPECT Imaging of the Encapsulated [^99m^Tc]TcO_4_^–^ Anion. J. Am. Chem. Soc..

